# Structural and functional analyses of Barth syndrome-causing mutations and alternative splicing in the tafazzin acyltransferase domain

**DOI:** 10.1016/j.mgene.2015.04.001

**Published:** 2015-04-22

**Authors:** Atsushi Hijikata, Kei Yura, Osamu Ohara, Mitiko Go

**Affiliations:** a Nagahama Institute of Bio-Science and Technology, 1266 Tamura-cho, Nagahama, Shiga 526-0829, Japan; b Graduate School of Humanities and Sciences, Ochanomizu University, 2-1-1 Otsuka, Bunkyo-ku, Tokyo 112-8610, Japan; c Laboratory for Integrative Genomics, RIKEN Center for Integrative Medical Sciences, 1-7-22 Suehiro-cho, Tsurumi-ku, Yokohama, Kanagawa 230-0045, Japan; d Department of Technology Development, Kazusa DNA Research Institute, 2-6-7 Kazusa-Kamatari, Kisarazu, Chiba 292-0818, Japan; e Research Organization of Information and Systems, 4-3-13, Toranomon, Minatoku, Tokyo 105-0001, Japan

**Keywords:** *TAZ* gene, X-linked recessive disease, Disease-causing mutations, Homology modeling, Immunodeficiency, Intrinsically unstructured region

## Abstract

Tafazzin is a mitochondrial phospholipid transacylase, and its mutations cause Barth syndrome (BTHS). Human tafazzin gene produces four distinct alternatively spliced transcripts. To understand the molecular mechanisms of tafazzin deficiency, we performed an atomic resolution analysis of the influence of the BTHS mutations and of alternative splicing on the structure and function of tafazzin. From the three-dimensional (3D) homology modeling of tafazzin, we identified candidate amino acid residues that contribute to cardiolipin binding and to mitochondrial membrane associations that facilitate acyl-transfer reactions. Primate specific exon 5, which is alternatively spliced, is predicted to correspond to an intrinsically unstructured region in the protein. We proposed that this region should change the substrate-binding affinity and/or contribute to primate-specific molecular interactions. Exon 7, another alternatively spliced exon, encodes a region forming a part of the putative substrate-binding cleft, suggesting that the gene products lacking exon 7 will lose their substrate-binding ability. We demonstrate a clear localization of the BTHS mutations at residues responsible for membrane association, substrate binding, and the conformational stability of tafazzin. These findings provide new insights into the function of defective tafazzin and the pathogenesis of BTHS at the level of protein 3D structure and the evolution of alternatively spliced exons in primates.

## Highlights

• We predicted a 3D structure model of tafazzin with reasonable reliability.
• The model structure accounts for differences in function of splice variants.
• The exon acquired in the primate lineage encodes an intrinsically unstructured region.
• The mutations associated with Barth syndrome were found on functionally important residues of tafazzin.

## Introduction

Tafazzin gene (*TAZ*) was originally identified as one of the genes responsible for Barth syndrome (BTHS, MIM# 302060), a severe X-linked disease characterized by mitochondrial cardiomyopathy, neutropenia, skeletal muscle weakness and growth retardation (Barth et al., 1983, Bione et al., 1996). *TAZ* gene encodes a phospholipid transacylase that is conserved in eukaryotes from yeast to humans (Neuwald, 1997, Xu et al., 2006). The human *TAZ* consists of 11 exons and produces four alternatively spliced mRNA transcripts: a full-length transcript containing all exons (FL), a transcript lacking exon 5 (Δ5), a transcript lacking exon 7 (Δ7) and a transcript lacking both exons 5 and 7 (Δ5Δ7) (Lu et al., 2004). Exon 5 was generated from a part of an intron during primate evolution within the hominoid lineage, and the alternative splicing of exons 5 and 7 has only currently been reported in humans (Gonzalez, 2005).

The tafazzin protein localizes to the mitochondrial membrane and is in charge of cardiolipin (CL) metabolism (Brandner et al., 2005). CL is a dimeric phospholipid containing four acyl-residues that is found exclusively on the mitochondrial inner membrane. The protein maintains the localization of mitochondrial proteins, including components of the respiratory chain and apoptosis regulatory proteins (Gonzalvez et al., 2008). BTHS patients with tafazzin mutations exhibit decreased CL levels, abnormal mitochondria, and deficiencies in mitochondrial respiratory chain function (Acehan et al., 2007). His69 and Asp74 in human tafazzin comprise an HX_4_D motif that is conserved and essential for the catalytic activity of the glycerolipid acyltransferase family (Heath and Rock, 1998). These two residues have been proposed to act as catalytic residues in tafazzin (Schlame, 2008), while no amino acid residues have been identified as being involved in CL binding.

A number of missense mutations in human *TAZ* have been identified in patients with BTHS, and this information is publicly available (https://www.barthsyndrome.org/). Recently, Claypool et al. (2011) examined the effects of BTHS mutations by introducing them into a tafazzin yeast homolog (Taz1) and demonstrated that these mutations affected the protein stability. For many other mutations, however, the degree to which they affect the molecular function of the tafazzin protein remains unclear.

Of the four alternatively spliced variants of human *TAZ*, FL and Δ5 encode proteins with transacylase activity, while Δ7 and Δ5Δ7 do not (Xu et al., 2009). The molecular basis for the variations in the functions of these splice variants is not well understood.

Knowledge of the 3D structure of tafazzin may provide insights into the mechanisms regarding how these disease-causing mutations and alternative splicing affect tafazzin function, however, the 3D structure of tafazzin has not been experimentally determined yet. When experimentally determined 3D structure is unavailable, homology modeling is a valuable method for obtaining structural and functional information for uncharacterized proteins (Baker and Sali, 2001). The 3D structure of a protein (target) can be built based on known 3D structures (templates) of homologous proteins (Chothia and Lesk, 1986). A template protein typically needs more than 30% sequence identity to the target protein. The closest related protein to tafazzin with a known 3D structure in a public database (http://pfam.sanger.ac.uk/) is, however, plant glycerol 3-phosphate acyltransferase (G3PAT) that has less than 20% sequence identity to human tafazzin. When the 3D structures of homologous proteins with low sequence identities are superimposed, there are often numerous insertions and deletions (indels) of amino acid residues. These indels of amino acid residues preclude the correct assignment of residue pairs in the target-template sequences through the use of conventional sequence alignment methods (Flores et al., 1993), hence difficulty in homology modeling arises. To overcome this difficulty, we developed ALAdeGAP, an improved amino acid sequence alignment method that utilizes a gap-penalty function reflecting the solvent accessibility of the amino acid residues in the 3D structure of the template protein. This new method yields better alignments than currently available methods for proteins with sequence identities as low as 20% (Hijikata et al., 2011). In this study, we built model structures of tafazzin using this new alignment method on the known structure of G3PAT. Based on this 3D model structure, we address the role of the acyltransferase domain in tafazzin function on a mitochondrial membrane. We proposed molecular mechanisms by which BTHS mutations and alternative splicing affect tafazzin protein function.

## Material and methods

### Search for the homologs of tafazzin and G3PAT sequences

The amino acid sequences of proteins homologous to human tafazzin (accession number: NP_000107.1) were obtained by searching the NCBI non-redundant protein sequence database (http://www.ncbi.nlm.nih.gov/, August 2008) using the BLAST program (Altschul et al., 1990) with a cutoff E-value of 10^− 14^. The amino acid sequences of proteins homologous to plant glycerol 3-phosphate acyltransferase (G3PAT) were also obtained in the same way.

### Prediction of the transmembrane region of tafazzin

The TOPCONS server (http://topcons.net/) was used to predict the location of the transmembrane helix in the tafazzin proteins. This server uses the consensus of the prediction results obtained from five distinct algorithms (Bernsel et al., 2009).

### Target-template sequence alignment

Two crystal structures of G3PAT from the cushaw squash are available in the Protein Data Bank (PDB, http://www.rcsb.org/). Their PDB IDs are 1k30 (Turnbull et al., 2001) and 1iuq (Tamada et al., 2004). Of these two structures, entry 1k30 was selected as a template for homology modeling, because it contained more atomic coordinates and had a reasonably high resolution. The amino acid sequences of tafazzin from humans, tafazzin from other eukaryotes and G3PAT were aligned using ALAdeGAP, an improved sequence alignment method, which utilizes the solvent accessibility of each amino acid residue in the template structure to calculate gap penalties (Hijikata et al., 2011).

### Homology modeling and model evaluation

The target-template sequence alignment was used to build a model structure of tafazzin with the MODELLER package (Marti-Renom et al., 2000). Structures were built for all tafazzin proteins in the multiple sequence alignment. For each tafazzin protein, 20 model structures were generated, which were assessed using the Discrete Optimized Protein Energy (DOPE) method implemented in the MODELLER package. The model with the best DOPE score was selected as the final model for each tafazzin protein. To assess the reliability of the final models, we used the ProSA-web server (https://prosa.services.came.sbg.ac.uk/prosa.php), a widely used tool that calculates a statistical potential derived from the atomic coordinates of protein structures deposited in PDB.

### Identification of the structural core and cleft

The structural cores of the proteins were determined as previously described (Yura and Go, 2008). Briefly, amino acid residues with solvent accessibilities less than 0.1 were defined as buried residues. If the buried residues were in contact with one another, they were defined as part of the protein core. The structural clefts of the analyzed proteins were determined using the CASTp server (http://sts-fw.bioengr.uic.edu/castp/).

## Results and discussion

### Target-template sequence alignment between tafazzin and G3PAT

Tafazzin belongs to a large protein superfamily, namely phospholipid acyltransferase including mitochondrial glycerol-3-phosphate acyltransferase, lysocardiolipin acyltransferases and so forth (Pfam ID: 01553). In this superfamily, G3PAT from cushaw squash is the only protein of which 3D structure was solved in atomic resolution (Tamada et al., 2004, Turnbull et al., 2001). G3PAT catalyzes the reaction of acyltransfer from acyl-CoA or acyl-acyl-carrier-protein (ACP) to glycerol-3-phosphate whereas tafazzin is known to catalyze a transacylation from phospholipid to lysophospholipid (Xu et al., 2009) without CoA or ACP. The difference in function of the proteins may cast a doubt using one of the protein structures as a template for building the other structure. However, in evolution of enzymes, there are many cases that enzymes in the same protein superfamily have different functions with the same sequence motifs and conserved structural core (reviewed in Galperin and Koonin, 2012). For example, exo-1,3-beta glucanase (EC: 3.2.1.58) from yeast and endo-beta-1,4 mannanase (EC: 3.2.1.78) from *Bacillus subtilis*, which belong to the same protein superfamily but apparently have different functions, have low sequence identity (18%) but their 3D structures are quite alike and the amino acid residues involved in the catalytic activity are highly conserved. This situation is quite alike to that of our case here and we built the structure of tafazzin catalytic domain structure based on the 3D structure of G3PAT from cushaw squash.

**Fig. 1:**
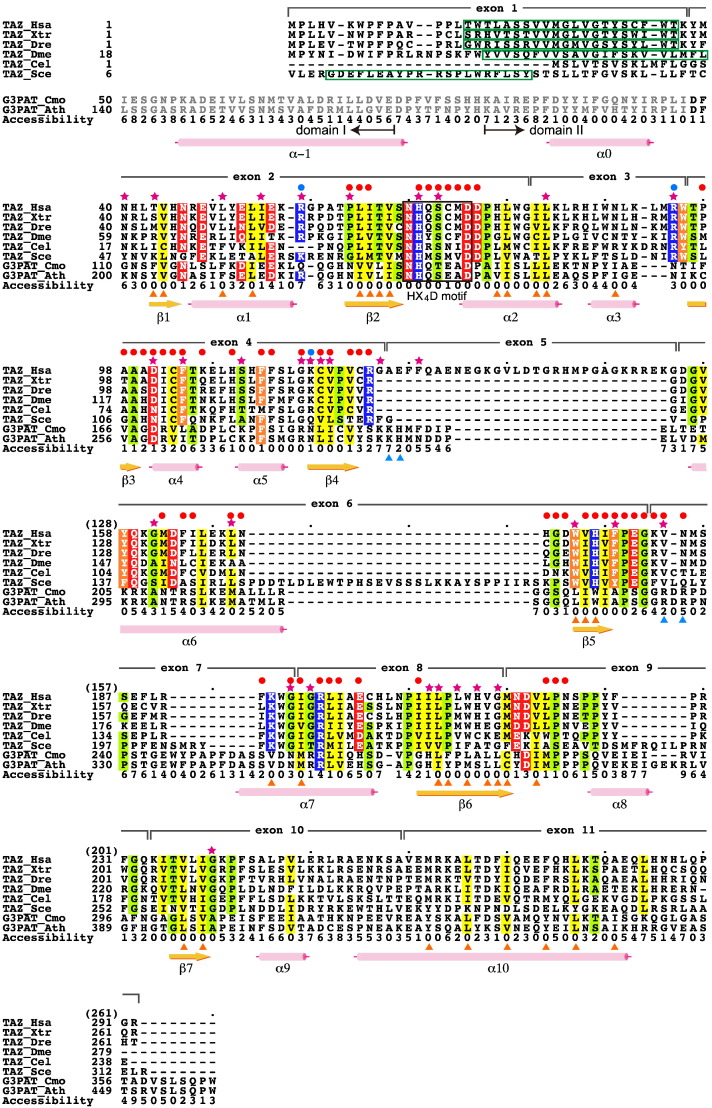
Multiple sequence alignment of tafazzin and G3PAT using ALAdeGAP. The secondary structural elements (α-helices and β-strands) and solvent accessibility normalized by 10 (0 to 9), which were derived from the crystal structure of G3PAT, are shown. The gray amino acid residues in G3PAT indicate regions structurally unrelated to tafazzin protein. The orange triangles indicate hydrophobic residues that form the structural core of G3PAT. The blue triangles indicate basic residues involved in the substrate binding of G3PAT. Amino acid positions are shaded when no less than 70% of the aligned residues are physicochemically similar: blue: H, K, and R; red: D, E, N, and Q; yellow: C, I, L, M, and V; green: A, G, P, S, and T; orange: F, W, and Y. The red filled circles indicate residues in the structural cleft of the human tafazzin model structure. The blue filled circles indicate positively charged residues that are predicted to be membrane associated. The purple stars indicate the sites of BTHS mutations. The exon boundaries of human tafazzin (TAZ_Hsa) are shown above the sequence. The number in a parenthesis in the TAZ_Hsa numbering is the residue number lacking exon 5, a deleted exon in a short isoform. The green boxes represent predicted transmembrane regions. The domain boundaries of G3PAT are indicated below the alignment. The abbreviation for each sequence identifier is as follows: Hsa, *Homo sapiens*; Xtr, *Xenopus tropicalis*; Dre, *Danio rerio*; Dme, *Drosophila melanogaster*; Cel, *Caenorhabditis elegans*; Sce, *Saccharomyces cerevisiae*; Cmo, *Cucurbita moschata* (cushaw squash); and Ath, *Arabidopsis thaliana*.

The X-ray crystal structure of cushaw squash G3PAT consists of two domains: domain I, an N-terminal four-helix bundle consisting of the first 77 residues, and domain II, a C-terminal catalytic acyltransferase domain with an α/β fold consisting of residues 85–384. A loop region, residues 78–84, connects the two domains (Turnbull et al., 2001). The sequence identity between the putative acyltransferase domain of human tafazzin (residues 41–245) and the sequence of domain II of G3PAT (residues 118–277) is only 16.3%. This low sequence identity made us use ALAdeGAP, which was shown to provide a reasonably reliable alignment in low sequence identity set (Hijikata et al., 2011), for obtaining a multiple sequence alignment between the tafazzin and G3PAT proteins (Fig. 1).

We confirmed the sequence alignment with two types of residue conservations at critically important sites for a globular enzyme, namely conservation at catalytic sites and at protein structural core formation sites (Go, 1983). In the sequence alignment, the HX_4_D motif, which is essential for the catalytic activity of glycerolipid acyltransferases including G3PAT and tafazzin (Heath and Rock, 1998), was perfectly aligned between the two family members (Fig. 1). The protein structural core of domain II of G3PAT consists of 33 hydrophobic residues of which 24 (73%) were aligned with hydrophobic residues of tafazzin (orange triangles in Fig. 1). These two types of conservations were consistent with the evolutionary conservation of the critical amino acid residues between these distantly related proteins, indicating that the overall 3D structures of acyltransferase domain of tafazzin and G3PAT would be similar enough to apply the homology modeling.

### Transmembrane region of tafazzin

Tafazzin is a membrane-associated protein that localizes to the mitochondrial intermembrane space. Two regions of yeast Taz1 were hypothesized to control Taz1 membrane association: the N-terminal 20 amino acid residues (Brandner et al., 2005) and the middle region of the acyltransferase domain (residues 215 to 232 of Taz1) (Claypool et al., 2006). We analyzed the tafazzin sequences from human, frog, zebrafish, fruit fly and yeast using TOPCONS, a method for predicting transmembrane helices, and only one transmembrane helix was consistently predicted in the N-terminal region, which is encoded by exon 1 in human (Fig. 1, green boxes). In contrast, G3PAT, a soluble protein localized to chloroplasts (Slabas et al., 2002), was predicted to possess no transmembrane helices. This result suggests that only the N-terminal region of tafazzin should form an α-helix, leading to membrane integration and anchoring.

### Model structure of Δ5 tafazzin

**Fig. 2:**
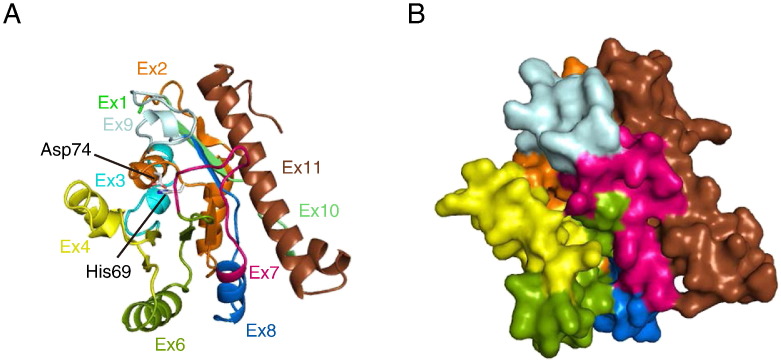
3D model structure of human Δ5 tafazzin. The structure is represented as (A) ribbon and (B) surface diagrams. The color corresponds to different exons. The conserved His and Asp residues in the HX_4_D motif, namely His69 and Asp74, are shown as stick diagrams in (A). This figure was generated using PyMOL (http://www.pymol.org/).

**Fig. 3:**
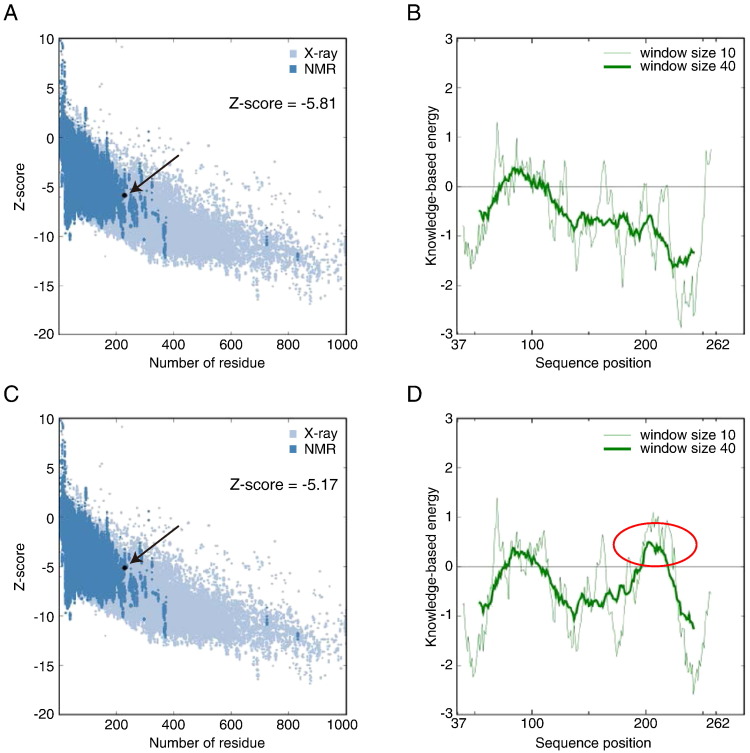
Model evaluation using ProSA. The quality plots of the model structures of human Δ5 tafazzin, which were generated based on ALAdeGAP (A) and ClustalW (C) alignments, are shown. The black dot indicates the Z-score of the model structure, and the blue background is the distribution of Z-scores for the experimentally determined structures. The residue-wise quality of the model structure is assessed and shown by plotting the energy against a residue number of the two models derived from the ALAdeGAP (B) and ClustalW (D) alignments. The horizontal axes in B and D indicate amino acid residue positions in the model structures of human Δ5 tafazzin. The red ellipse in D indicates a region of low quality in the model.

The sequence alignment between the tafazzin and G3PAT proteins showed no template structure corresponding to a region encoded by exon 5 in humans (Fig. 1). Using the 3D structure of domain II of G3PAT, we generated a 3D structure model of Δ5 tafazzin (lacking exon 5) (Fig. 2), which encodes one of the enzymatically active proteins (Xu et al., 2009). To evaluate the reliability of the model structure, we used the ProSA-web server (https://prosa.services.came.sbg.ac.at/prosa.php) and found that the Z-score of the human Δ5 tafazzin structure was − 5.81. This value is consistent with the Z-score distribution of experimentally determined structures in the PDB (Fig. 3A and B). For comparison, we built a multiple sequence alignment by ClustalW (Thompson et al., 1994), a widely employed alignment tool, and generated a 3D structure model. The Z-score of this structure was worse than that by ALAdeGAP alignment (Fig. 3C and D). When the two homology-modeled structures were compared, a large difference was noted in the region containing the β7 strand (Supplementary Fig. S1). In the Δ5 tafazzin model structure based on the ClustalW alignment, the basic residue Lys211 was buried in the interior of the protein, and the hydrophobic residues Ile205, Val207 and Ile209 were exposed to solvent. On the contrary, these residues were located in appropriate locations in the model structure by ALAdeGAP alignment. Lys211 was located on the surface, and Ile205, Val207 and Ile209 were buried inside the protein (Fig. 3B and D). The observed differences between these model structures are consistent with their ProSA scores. In globular proteins, hydrophilic residues tend to be exposed on the surface, and hydrophobic residues are buried in the interior of proteins (Go and Miyazawa, 1980). Thus, of the two model structures, ALAdeGAP model structure of tafazzin is much consistent with our current picture of a globular protein structure.

### Putative phospholipid-binding site of tafazzin

**Fig. 4:**
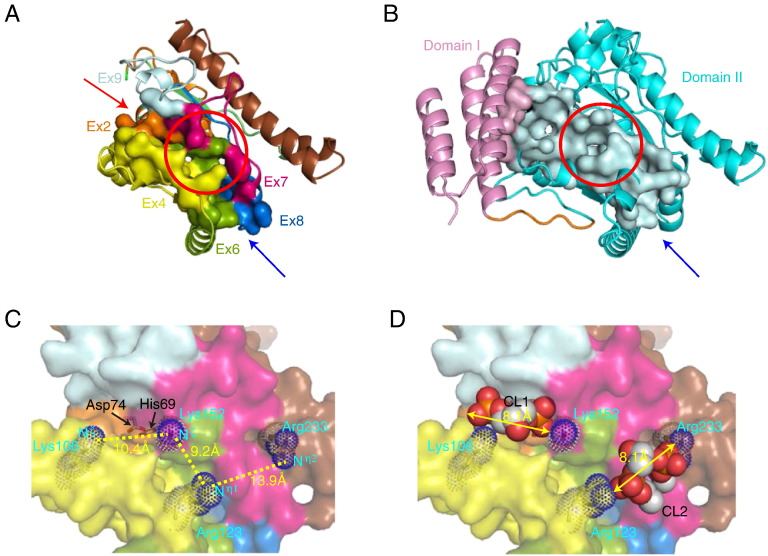
Structural clefts in tafazzin and G3PAT. Clefts identified in the structure of (A) human Δ5 tafazzin and (B) G3PAT are shown. The atoms in the cleft are indicated by surface representation diagram. The red and blue arrows indicate the open ends of the structural clefts. The red circles indicate the structural positions where positively charged residues are located. (C) An enlarged view of the putative substrate-binding site of human Δ5 tafazzin. His69 and Asp74 in the HX_4_D motif are depicted as stick diagrams. The four positively charged residues located in the cleft are depicted as blue dot diagrams. (D) Proposed substrate-binding model of human Δ5 tafazzin. The head groups of two CL molecules are depicted as space-filling models. The coloring of the amino acid residues is the same as that of Fig. 2.

Catalytic residues in tafazzin have been proposed by sequence similarity; however, no amino acid residues involved in substrate binding have been characterized. Tafazzin catalyzes reactions that transfer an acyl chain between two dimeric phospholipid molecules, CL and monolyso-CL (MLCL) (Malhotra et al., 2009). On the other hand, G3PAT catalyzes the acylation of glycerol 3-phosphate (G3P) using an acyl-carrier protein as the acyl donor (Murata and Tasaka, 1997). The crystal structure of G3PAT contains a large open cleft that accommodates the fatty acyl substrate at one end, while the other end is blocked by domain I (Tamada et al., 2004). We assigned a putative phospholipid-binding cleft in the model structure of human Δ5 tafazzin and characterized 57 amino acid residues as ones forming the cleft (Fig. 4A). This area encompasses residues encoded by six exons (exons 2, 4, 6, 7, 8 and 9), and more than half of these residues (33 out of 57 residues) are located in regions encoded by either exon 4 or 6 (Table 1). The putative substrate-binding cleft of tafazzin differs from that of G3PAT in terms of the degree of openness, i.e., tafazzin has the cleft with two open ends, whereas G3PAT has the cleft with only one open end (shown by red and blue arrows in Fig. 4A and B, respectively). The distinct architecture in Δ5 tafazzin is due to the lack of a region in amino acid sequence corresponding to domain I of G3PAT. The difference well correlates with the difference in the structures of the substrate molecules between tafazzin and G3PAT, i.e., both the acyl donor (CL) and acyl acceptor (MLCL) of tafazzin contain long acyl chains, whereas the acyl acceptor (G3P) of G3PAT does not.

The positively charged residues required for the binding of a G3P phosphate group are located in the cleft of the G3PAT structure (Slabas et al., 2002). These residues are not conserved in tafazzin proteins (Fig. 1). However, on the Δ5 tafazzin model structure, there exists a patch of conserved positively charged residues in the cleft of tafazzin. Lys106, Arg123, Lys152 and Arg233 line the putative substrate-binding cleft, and the catalytic residues His69 and Asp74 are located behind these positively charged residues (Fig. 4C). The relative location of the four positively charged residues against the catalytic sites allowed us to dock a CL molecule smoothly. When 78 CL molecules in Het-PDB Navi (Yamaguchi et al., 2004) were examined, the average distance between the oxygen atoms in two CL phosphate groups was 8.1 Å (standard deviation is 0.4 Å). In our model structure, the distances between the amino groups of the basic residues were between 9.2 Å and 13.9 Å (Lys106 Nζ–Lys152 Nζ = 10.4 Å, Lys152 Nζ–Arg123 Nη1 = 9.2 Å, Arg123 Nη1–Arg233 Nη2 = 13.9 Å) (Fig. 4C), the distances enabling reasonable placement of CL. The placement of the CL head groups on the tafazzin model structure indeed demonstrated that the two basic residue pairs, Lys106–Arg123 and Lys152–Arg233, could interact with the phosphate groups of CL (Fig. 4D). The similar mode of interactions was found, for instance, in a crystal structure of yeast cytochrome *bc1* complexed with CL (PDB code: 1kb9). The amino groups of two lysine residues (Lys228 and Lys289) are separated by approximately 11 Å and make contact with two phosphate groups present in the CL molecule (Lange et al., 2001). Our model structure consistently explained the mode of interactions between tafazzin and CL/MLCL molecules.

### Functional assessment of alternative splicing of exons 5 and 7 of human TAZ

Four splicing variants of human *TAZ* differ in the inclusion or exclusion of exons 5 and 7. The splicing variants FL and Δ5 *TAZ* encode proteins that have transacylase activity, whereas the splicing variants Δ7 and Δ5Δ7 *TAZ* do not (Xu et al., 2009). In our model structure of Δ5 tafazzin, the amino acid residues encoded by exon 7 were involved in the formation of the putative substrate-binding cleft (Table 1) and formed a portion of the structural core of the protein (Supplementary Fig. S2). The lack of function in Δ7 variants could be attributed to the disruption of both the substrate-binding cleft and the structural core of the protein. We have previously found many cases of exon-skipping type of alternative splicing that putatively disrupt function of the protein (Yura et al., 2006) and the splicing variants Δ7 *TAZ* seems to be another example of this case.

Exon 5 encodes 30 amino acid residues, from 125 to 154 (Fig. 1), and is found exclusively in *TAZ* genes of the hominoid primate lineage (Gonzalez, 2005). This amino acid sequence is highly conserved in primates; however its functional role has not been characterized yet. The lack of a template structure corresponding to the region encoded in the human exon 5 makes it difficult to build a 3D structure model for this region (Fig. 1). In addition, hydrophilic and charged amino acid residues comprise a large portion of the sequence encoded by exon 5, suggesting that this region does not have a stable compact 3D structure. When we predicted the intrinsically unstructured regions present in human tafazzin using 15 available prediction servers, eleven of them consistently predicted that the region encoded by exon 5 is intrinsically unstructured, and no other regions of the tafazzin sequence were consistently predicted as intrinsically unstructured (Supplementary Table S1). The addition of this putative unstructured amino acid stretch encoded by exon 5 onto the model structure of Δ5 tafazzin would unlikely disturb the overall 3D structure of the tafazzin acyltransferase domain, because Δ5 tafazzin model structure showed that location of the insertion, namely between the amino acid residues encoded by the 3′ end of exon 4 and the 5′ end of exon 6, is exposed on the surface of the model (Fig. 2). Thus FL tafazzin likely has similar structure to Δ5 tafazzin model structure plus the unstructured protrusion. The predicted stable conformation of this FL tafazzin provides a structural basis for the report that FL tafazzin functions as a phospholipid transacylase (Xu et al., 2009).

Intrinsically unstructured regions in proteins are often involved in molecular interactions (Liu et al., 2009, Yura and Hayward, 2009). For example, human pyrophosphorylase contains a 17-residue long unstructured stretch which contributes to modulations of the protein's oligomeric assembly and active site architecture. This stretch is a target of alternative splicing (Peneff et al., 2001). A recent study shows that evolutionarily conserved unstructured regions in human proteins tend to be spliced as a unit by alternative splicing events, indicating that these regions can be attached and detached as functional modular units (Pentony and Jones, 2010). The region encoded by exon 5 of tafazzin seems to be the same type of functional unit that modulates the substrate-binding specificity and/or affinity, or that contributes to its interactions with other molecules in a hominoid primate-specific manner. Exon 5 is thought to arise from the conversion of a part of an intron to an exon (Gonzalez, 2005). In the discovery stage of introns in eukaryotes, it was reported that exon corresponds to one or more compact modules of proteins (Go, 1981). Thus, we speculate that this alternatively spliced exon 5 of the *TAZ* gene is at the early stage of creating new networks within the molecules in cells through its flexible conformations and that such flexible conformations may be fixed into a compact module structure in the process of adaptation to a specific network during the evolutionary time scale.

### Association of tafazzin with the mitochondrial inner membrane

We predicted that tafazzin contains a transmembrane helix at its N-terminus (Fig. 1), and our model supports the hypothesis by Brandner et al. (2005) that the helix integrates into the mitochondrial membrane. Claypool et al. (2006) suggested another hypothesis for the membrane association that the acyltransferase domain of tafazzin itself associates with the mitochondrial membrane. Based on our model structure, we examined whether the tafazzin acyltransferase domain itself has a potential to associate with the mitochondrial inner membrane.

**Fig. 5:**
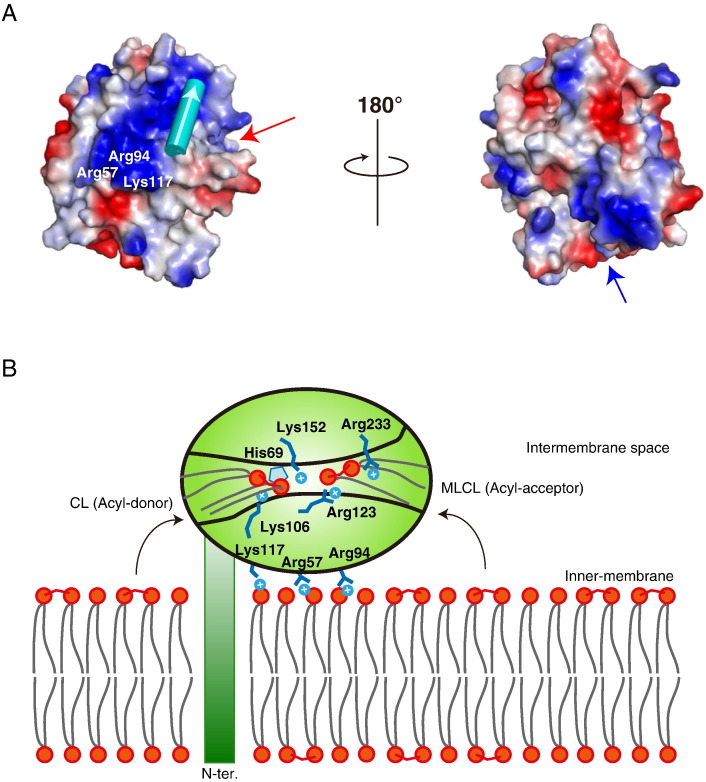
Surface electrostatic potential and a proposed membrane association model. (A) Surface models of human Δ5 tafazzin. The red and blue arrows indicate the open ends of the structural clefts shown in Fig. 4. The cylinder in cyan represents the proposed position of the N-terminal transmembrane α-helix, and the white arrow indicates the direction (N- to C-terminus) of the helix. The electrostatic potential in vacuum was calculated using the PyMOL package. (B) Schematic diagram of a membrane localization model of tafazzin. The tafazzin protein (green oval) associates with the mitochondrial inner membrane through the transmembrane helix in the N-terminus and the positively charged residues (Arg57, Arg94 and Lys117) on the protein surface near the putative substrate-binding cleft. Other positively charged residues (Lys106, Arg123, Lys152 and Arg233) in the cleft interact with the phosphate groups of the CL and MLCL acyl-donor and -acceptor, recruiting them into the catalytic site (His69).

The model structure of human Δ5 tafazzin has a net positive charge of 5.0. We confirmed that the positive electrostatic potential on the surface of tafazzin is conserved in homologous proteins, i.e., zebrafish (net positive charge = 3.0) and fruit fly (net positive charge = 7.0) (Supplementary Fig. S3). An electrostatically positive patch composed of Arg57, Arg94 and Lys117 is present on the surface of human tafazzin close to the putative substrate-binding cleft (Fig. 5A). These residues are conserved among the tafazzin homologs (Fig. 1), and the electrostatic patch is located near the N-terminal region predicted to form the transmembrane helix (Fig. 5A). In contrast, the structure of G3PAT, which does not associate with the membrane, does not have such a positive patch (Supplementary Fig. S3). In many membrane-associated proteins, conserved positive patches facilitate membrane binding through electrostatic interactions (Murray et al., 2005). Given that tafazzin localizes to the mitochondrial membrane, these conserved positively charged residues could be important for mediating interactions with the negatively charged phospholipid head groups of the mitochondrial membrane, which is consistent with other membrane-associated enzymes that act on lipophilic molecules (Forneris and Mattevi, 2008). This association mechanism brings the substrate-binding cleft closer to the membrane surface where the substrate molecules are located (Razeto et al., 2007).

Our model suggests that the hypotheses by both Brandner et al. (2005) and Claypool et al. (2006) can be realized in a consistent manner. The modeled tafazzin protein can associate with the mitochondrial membrane through its single N-terminal transmembrane helix and through the surface-exposed positive patch to accomplish its transacylase activity (Fig. 5B). In our model, the electrostatic interactions between tafazzin and phospholipids in the mitochondrial membrane place the substrate-binding cleft closer to the membrane surface, allowing the cleft to gain access to the phosphate groups of the dimeric phospholipids that act as acyl-donor and -acceptor and enabling the easy recruitment of the phospholipid substrates into the catalytic site of tafazzin (Fig. 5B).

### Disease-causing mutations located at functionally relevant sites in tafazzin

**Fig. 6:**
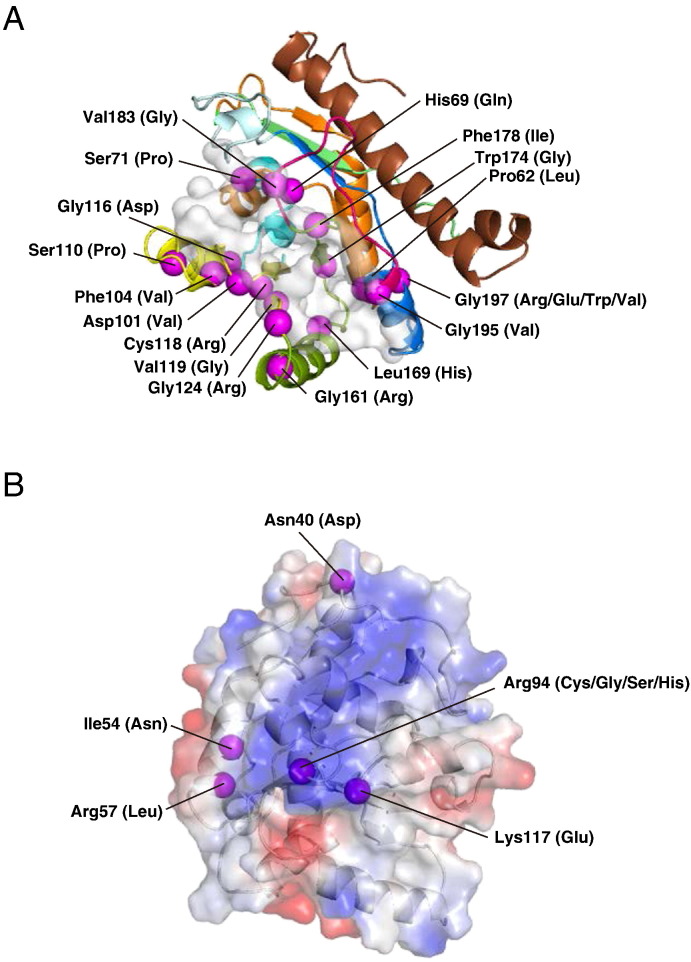
Locations of disease-causing missense mutations in the tafazzin model structure. Mutation positions in the putative substrate-binding cleft (A) and the positively charged surface patch proposed as the interaction region with the membrane are indicated (B). Mutations in buried positions are not shown. The positions of residues mutated in patients with BTHS are represented as purple spheres. The amino acids in parentheses indicate substituted residues found in affected patients.

Thirty-nine disease-causing missense mutations have been identified from BTHS patients at 33 unique residue positions in human tafazzin (Table 2). The positions of mutated residues spread across eight exons (Fig. 1). Exon 2 and exon 4 contain the highest number of mutation positions (8 sites) followed by exon 8 (6 sites) and exon 6 (4 sites). We mapped the mutation positions on the tafazzin model structure and found that each residue was located at one of the following three regions of the model structure: 1) the putative substrate-binding cleft or catalytic site, 2) the predicted membrane-associated region on the protein surface, and 3) the buried region. Half of the 33 mutated residue positions, namely Pro62, His69, Ser71, Asp101, Phe104, Ser110, Gly116, Cys118, Val119, Gly124, Gly161, Leu169, Trp174, Phe178, Val183, Gly195 and Gly197, were located at or close to the putative substrate-binding cleft (Fig. 6A). His69 is thought to be the catalytic site for the transacylase activity (Heath and Rock, 1998). Thus, the His69Gln mutation would directly disrupt the enzymatic activity of tafazzin. The Gly116Asp, Cys118Arg, Gly124Arg, Gly161Arg, Leu169His, Gly197Arg and Gly197Glu mutations change non-polar amino acid residues to charged ones within the putative binding cleft. The Pro62Leu, Phe104Val, Gly197Trp and Gly197Val mutations either reduce or augment the volume of the cleft, and these mutations could directly affect substrate binding by altering the conformation of the cleft. Additionally, Ser71Pro, Asp101Val and Ser110Pro mutations lead to the loss of polar side chains, and likely to the loss of the catalytic function through the disruption of hydrogen bonds or electrostatic interactions involved in substrate binding.

Three positively charged residues, Arg57, Arg94 and Lys117, are suggested to be involved in the association of tafazzin with the mitochondrial membrane (Fig. 5B). In patients with BTHS, several amino acid substitutions have been found that result in the loss or weakening of the positive charge (Arg57Leu, Arg94Cys, Arg94Gly, Arg94Ser, Arg94His and Lys117Glu; Fig. 6B). These six mutations could reduce the positive electrostatic potential on the surface of tafazzin, leading to a decrease in association with the mitochondrial membrane and to a lower chance of substrate binding as a result. Two other residues mutated in BTHS, Asn40 and Ile54, are also located on the surface close to the positively charged patch (Fig. 6B). The Asn40Asp mutation introduces a negatively charged residue on the surface of tafazzin, which likely influences the positively charged patch and reduces the interactions with the membrane. Ile54 is located on helix α1 and makes hydrophobic contact with the side chain of Tyr51. The Ile54Asn mutation could break this hydrophobic interaction, change the local conformation of helix α1, and finally affect the protein–membrane interaction. Eight of the mutations mentioned above are related to a loss or reduction of interactions with the membrane.

Thr43, Leu50, Gly80, Leu82, Ile209, Leu210, Leu212, His214, Gly216 and Gly240 are located in secondary structures (helices α1, α2 and strands β6, β7) and are buried inside of tafazzin, making up the core structure of the protein (Fig. 1 and Table 2). The mutations Gly80Glu, Ile209Asp, Leu210Arg, His214Arg, Gly216Arg and Gly240Arg are substitutions for charged amino acids. Such substitutions within the protein core are known to destabilize the protein conformation. In patients with BTHS, Pro is substituted with Thr43, Leu50, Leu82 and Leu212. Substitutions of residues in α-helices or β-strands with Pro are known to partially break secondary structures, because Pro does not possess an amide group that can donate a hydrogen atom to the carbonyl group of another residue. The partial deformation of the secondary structure due to a mutation within the *TAZ* gene is expected to reduce protein stability of tafazzin. A recent study demonstrated that mutant yeast Taz1 protein with an Ala88Glu, Ser140Arg or Leu148His mutation (equivalent to Gly80Glu, Gly161Arg or Leu169His in BTHS patients, respectively) expressed at low levels is degraded by the intermembrane space AAA protease, suggesting a low stability or unfolding of the mutant proteins (Claypool et al., 2011). Our model structure accounts for the experimental observations.

Phe128Ser is the only known mutation in exon 5. Exon 5 is dispensable for tafazzin function. Thus, it is puzzling that a mutation of this exon affects the protein function. Indeed, the pathogenicity of this mutation in BTHS is still controversial (Xu et al., 2009), and it has been reported that the Phe128Ser mutation is more likely a rare polymorphism restricted to African American populations and has little relation to disease (Taylor et al., 2009). We predicted that the region encoded by exon 5 is intrinsically unstructured. We hence speculate that this region facilitates substrate binding or interactions with unknown proteins. The Phe128Ser mutation could affect interactions with other molecules to some extent due to a change in the side chain volume from bulky to small. Further investigations are required to evaluate the effect of this mutation.

Taken together, our model provides structural basis for the altered function of tafazzin that has been observed in BTHS patients with CL metabolism defects.

## Conclusion

We built a 3D structure model of the tafazzin protein using the new sequence alignment method that improves the alignment quality when the sequence identity is low. The generated model structure was applied to identify structurally and functionally relevant amino acid residues of tafazzin. The atomic data provide new insights into the interaction of tafazzin with the membrane and the substrate-binding cleft. The model was also applied to analyze the molecular mechanisms of BTHS pathogenesis that have not been characterized. Based on the 3D structure of tafazzin protein, BTHS pathogenesis was found related to substrate binding, catalytic activity, membrane association and the reduction in the conformational stability. In addition, the regulatory role of alternative splicing in substrate binding is proposed. This 3D structure of tafazzin can be utilized as a model to predict the effects of mutations of the tafazzin protein and may help provide the prognosis of BTHS through the use of personal genome analyses.

## Figures and Tables

**Table 1:** Distribution of the amino acid residues forming the substrate-binding cleft.

Exon no.	Residue counts^a^	Fraction (%)
2	10	17.5
4	18	31.6
6	15	26.3
7	5	8.8
8	6	10.5
9	3	5.3
Total	57	100.0

a The number of the cleft-forming residues encoded by each exon.

**Table 2:** Proposed functional affects of the disease-causing missense mutations in tafazzin.

Mutations^a^	Exon	Location on 3D structure	Electrostatic interactions	Conformational stability	Proposed functional effects
p.Asn40Asp	2	Surface	X		Membrane association
p.Thr43Pro	2	Buried		X	Destabilization
p.Leu50Pro	2	Buried		X	Destabilization
p.Ile54Asn	2	Surface		X	Membrane association
p.Arg57Leu	2	Surface	X		Membrane association
p.Pro62Leu	2	Cleft		X	Substrate binding
p.His69Gln	2	Catalytic site	X		Catalytic activity
p.Ser71Pro	2	Cleft		X	Substrate binding
p.Gly80Glu	2	Buried	X	X	Destabilization
p.Leu82Pro	3	Buried		X	Destabilization
p.Arg94Cys	3	Surface	X		Membrane association
p.Arg94Gly	3	Surface	X		Membrane association
p.Arg94Ser	3	Surface	X		Membrane association
p.Arg94His	3	Surface	X		Membrane association
p.Asp101Val	4	Cleft	X	X	Substrate binding
p.Phe104Val	4	Cleft		X	Substrate binding
p.Ser110Pro	4	Cleft		X	Substrate binding
p.Gly116Asp	4	Cleft	X	X	Substrate binding
p.Lys117Glu	4	Surface	X		Membrane association
p.Cys118Arg	4	Cleft	X	X	Substrate binding
p.Val119Gly	4	Cleft		X	Substrate binding
p.Gly124Arg	4	Cleft	X	X	Substrate binding
p.Phe128Ser	5	Cleft^b^		X	Substrate binding
p.Gly161Arg	6	Cleft	X	X	Substrate binding
p.Leu169His	6	Cleft	X		Substrate binding
p.Trp174Gly	6	Cleft		X	Substrate binding
p.Phe178Ile	6	Cleft		X	Substrate binding
p.Val183Gly	7	Cleft		X	Substrate binding
p.Gly195Val	7	Cleft		X	Substrate binding
p.Gly197Arg	8	Cleft	X	X	Substrate binding
p.Gly197Glu	8	Cleft	X	X	Substrate binding
p.Gly197Trp	8	Cleft		X	Substrate binding
p.Gly197Val	8	Cleft		X	Substrate binding
p.Ile209Asp	8	Buried	X	X	Destabilization
p.Leu210Arg	8	Buried	X	X	Destabilization
p.Leu212Pro	8	Buried		X	Destabilization
p.His214Arg	8	Buried	X	X	Destabilization
p.Gly216Arg	8	Buried	X	X	Destabilization
p.Gly240Arg	10	Buried	X	X	Destabilization

a The notation of each amino acid substitution follows the recommendation provided by the Human Gene Variation Society (http://www.hgvs.org/).

b The 3D model structure of this residue was not generated due to a lack of the corresponding template structure. The 3D position is inferred from the structural context (see Disease-causing mutations located at functionally relevant sites in tafazzin in the main text).

## Appendix A Supplementary data

Fig. S1. Multiple sequence alignment of tafazzin and G3PAT with the ClustalW program. The representation follows Fig. 1. See text for the red box. Fig. S2. Structural core in the human Δ5 tafazzin model structure. The model structures of human Δ5 tafazzin (A) and Δ5Δ7 tafazzin (B) are colored by exons. The amino acid residues forming structural core of Δ5 tafazzin (C) and Δ5Δ7 tafazzin (D) are colored in yellow. The region colored magenta on the Δ5 tafazzin model indicates the residues encoded by exon 7. (E) Distribution of the number of amino acid residues forming the structural core to different exons in the human Δ5 tafazzin model. Fig. S3. The electrostatic potential on the surface of the tafazzin homologs and G3PAT structures. The surface model of (A) *Danio rerio* and (B) *Drosophila melanogaster*, respectively, and (C) G3PAT in *Cucurbita moschata* is presented. The vacuum electrostatic potential was calculated using the PyMOL package. The orientations of model structures are the same as in Fig. 5.

Table S1 Predicted disordered regions in the human tafazzin.
