# Supplementary figures and images for: Structural and functional analyses of Barth syndrome-causing mutations and alternative splicing in the tafazzin acyltransferase domain
Source: Meta Gene. 2015 Apr 22;4:92–106. doi: 10.1016/j.mgene.2015.04.001 (PMC4412953; doi:10.1016/j.mgene.2015.04.001)

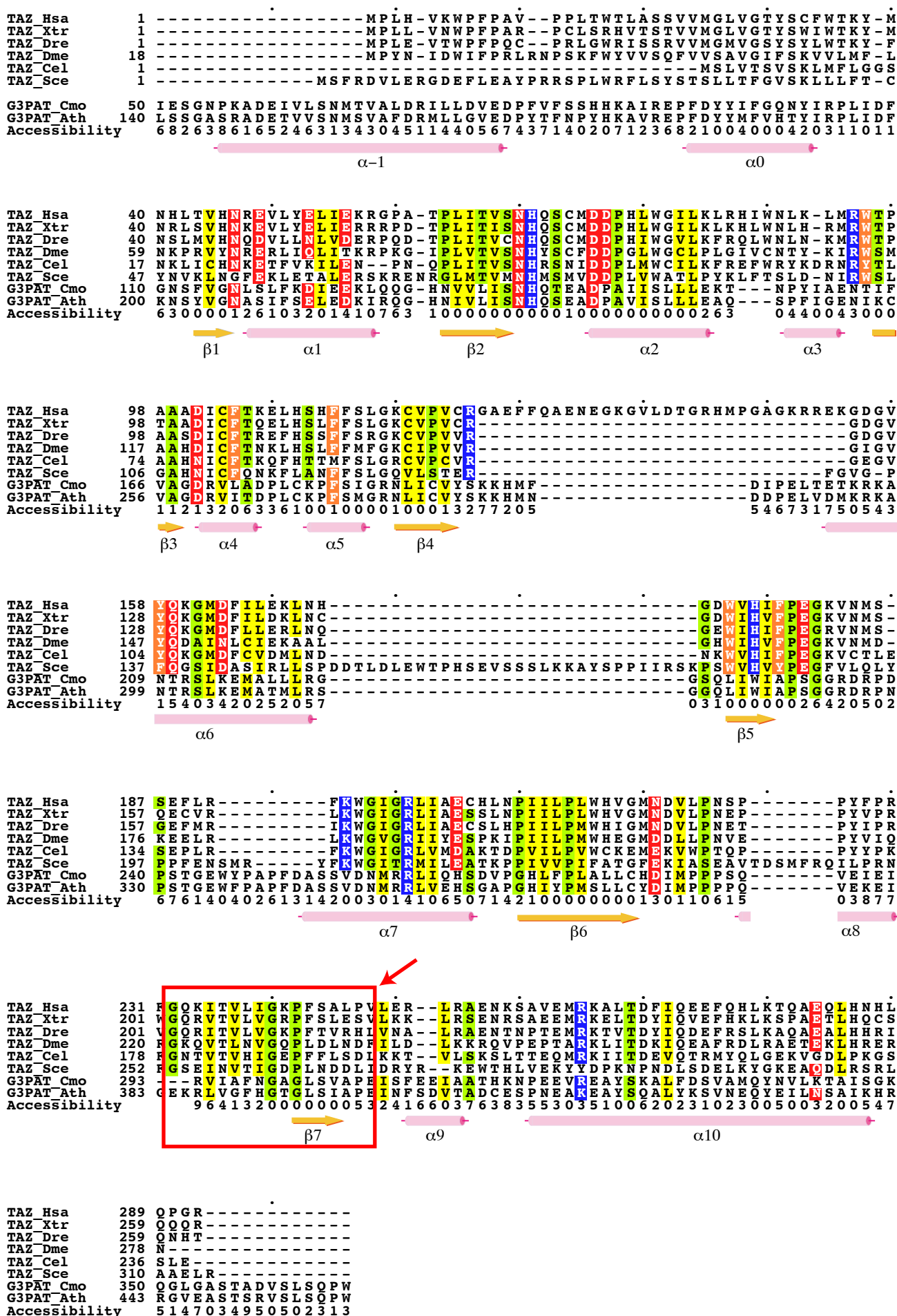

Supplementary figure S1

A

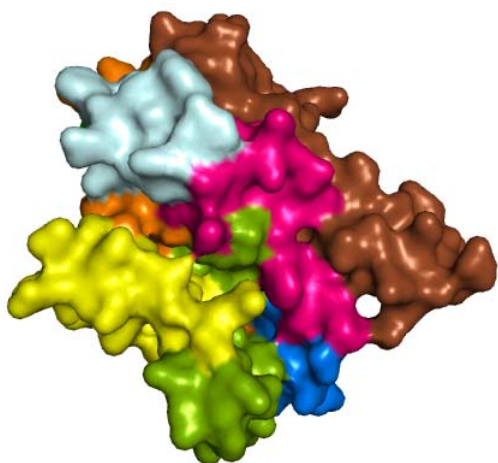

B

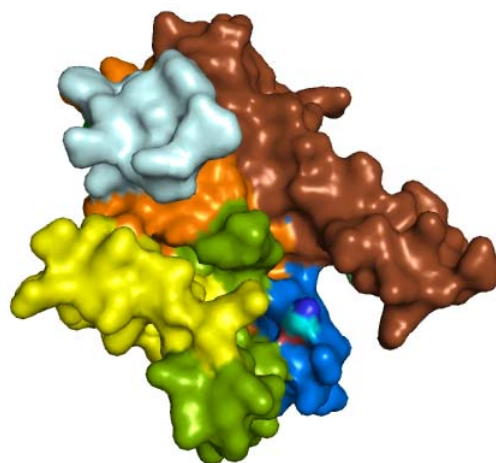

C

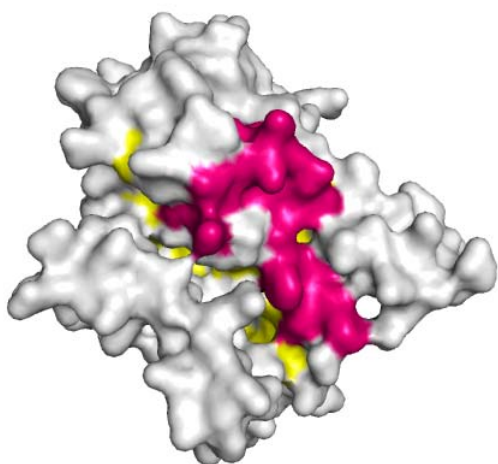

D

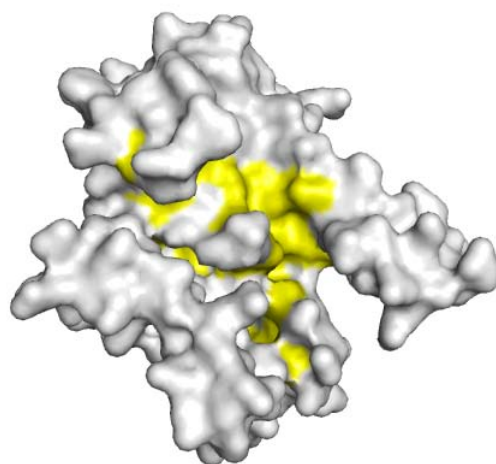

E

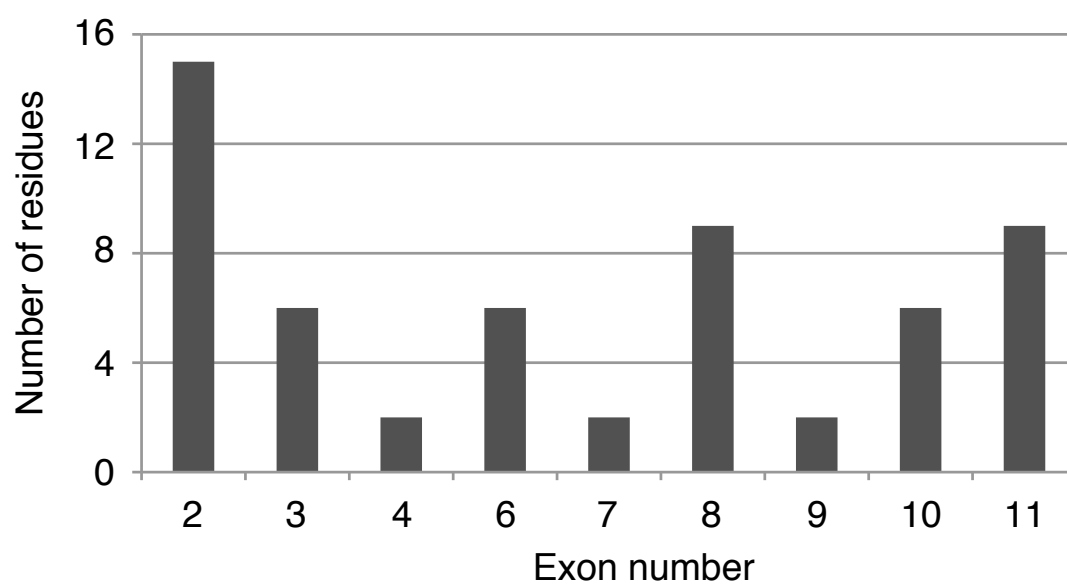

A

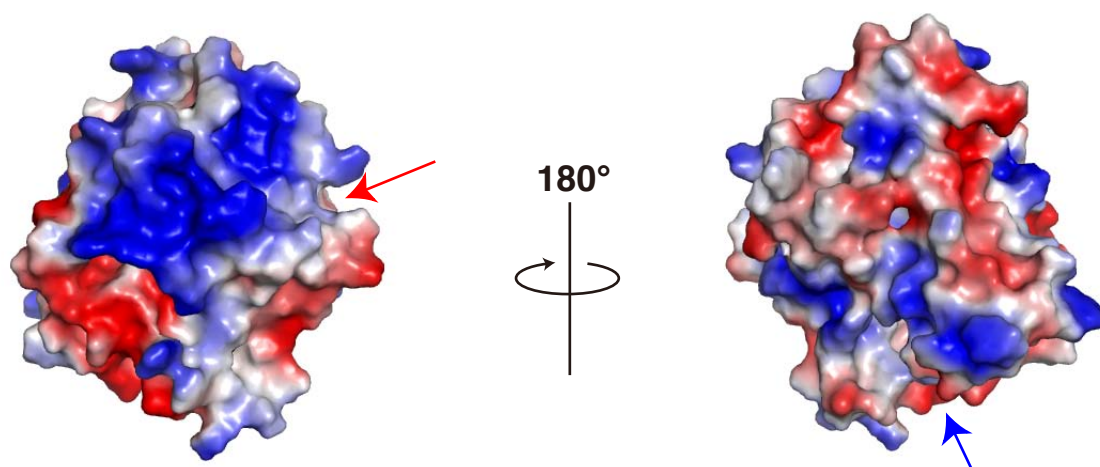

B

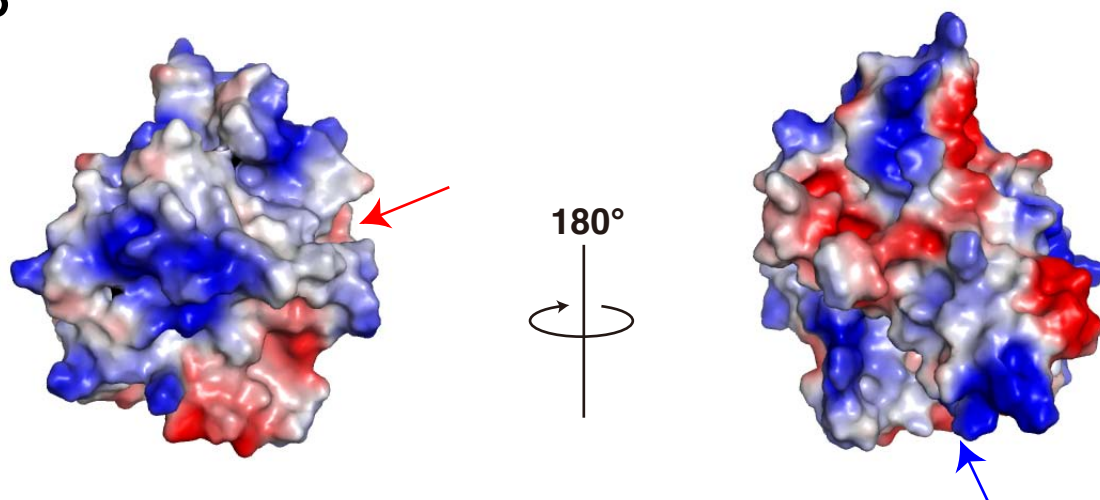

C

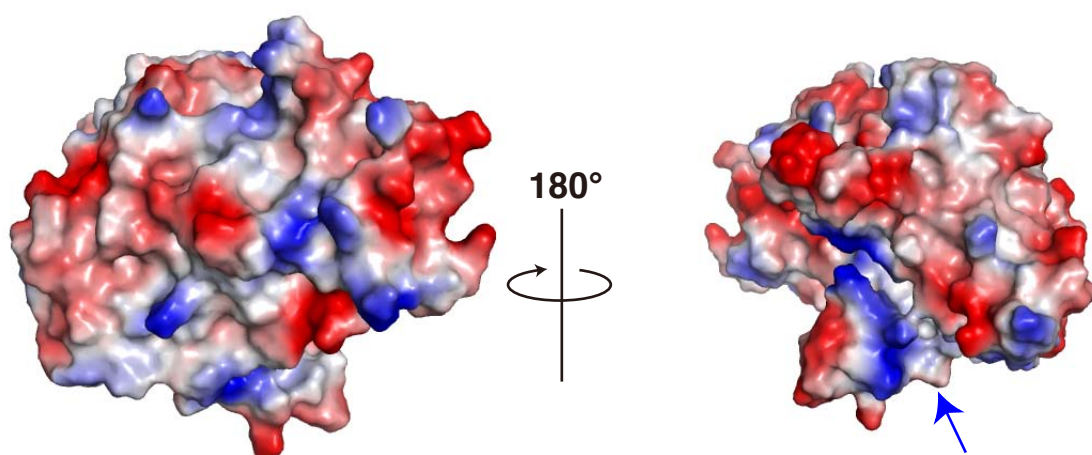

Supplement: Supplementary file 1 — Fig. S1. Multiple sequence alignment of tafazzin and G3PAT with the ClustalW program. The representation follows Fig. 1. See text for the red box. Fig. S2. Structural core in the human Δ5 tafazzin model structure. The model structures of human Δ5 tafazzin (A) and Δ5Δ7 tafazzin (B) are colored by exons. The amino acid residues forming structural core of Δ5 tafazzin (C) and Δ5Δ7 tafazzin (D) are colored in yellow. The region colored magenta on the Δ5 tafazzin model indicates the residues encoded by exon 7. (E) Distribution of the number of amino acid residues forming the structural core to different exons in the human Δ5 tafazzin model. Fig. S3. The electrostatic potential on the surface of the tafazzin homologs and G3PAT structures. The surface model of (A) Danio rerio and (B) Drosophila melanogaster, respectively, and (C) G3PAT in Cucurbita moschata is presented. The vacuum electrostatic potential was calculated using the PyMOL package. The orientations of model structures are the same as in Fig. 5. [file mmc1.pdf]
