# Supplementary material for: Structural and functional analyses of Barth syndrome-causing mutations and alternative splicing in the tafazzin acyltransferase domain
Source: Meta Gene. 2015 Apr 22;4:92–106. doi: 10.1016/j.mgene.2015.04.001 (PMC4412953; doi:10.1016/j.mgene.2015.04.001)
Supplement: Table S1 — Predicted disordered regions in the human tafazzin. [file mmc2.doc]

**Supporting information/Supplementary material**

**Table S1.**

Predicted disordered regions in the human tafazzin.

**Server name Predicted regions URL Reference**

Poodle-I 136-156; 252-263; 287-292 http://mbs.cbrc.jp/poodle/index.html Shimizu *et al*., 2007

PrDOS 1-3; 131-158; 281-292 http://prdos.hgc.jp Ishida *et al*., 2007

iPDA 139-160; 260-292 http://biominer.bime.ntu.edu.tw/ipda/ Su *et al*., 2007

FoldUnfold 128-141; 143-158 http://skuld.protres.ru/~mlobanov/ogu/ogu.cgi Galzitskaya *et al*., 2006

SPRITZ 131-135; 139-152 http://protein.cribi.unipd.it/spritz/ Vullo *et al*., 2006

DISpro Not predicted http://www.ics.uci.edu/%7Ebaldig/scratch/ Cheng *et al*., 2005

FoldIndex 133-140; 144-173 http://bip.weizmann.ac.il/fldbin/findex Prilusky *et al*., 2005

Iupred 138-155; 281-292 http://iupred.enzim.hu/index.html Dosztanyi *et al*., 2005

RONN 3-21; 130-151; 246-263 http://www.strubi.ox.ac.uk/RONN Yang *et al*., 2005

DISPROT 132-137; 140-144; 148-153; 286-292 http://www.ist.temple.edu/disprot/Predictors.html Peng *et al*., 2005

PreLink Not predicted http://genomics.eu.org/prelink/ Coeytaux *et al*., 2005

DISOPRED2 281-292 http://bioinf.cs.ucl.ac.uk/disopred/disopred.html Ward *et al*., 2004

DisEMBL 1.5 1-13; 129-156; 284-292 http://dis.embl.de/ Linding *et al*., 2003

NORSp Not predicted http://cubic.bioc.columbia.edu/services/NORSp/ Liu *et al*., 2003

GlobPlot 2.3 7-11; 66-74; 136-154; 217-230 http://globplot.embl.de/ Linding *et al*., 2003

Predicted region with underline indicates that the region contains amino acid residues encoded by exon5 (124-154) in the human tafazzin.

**References for intrinsically disorder prediction methods**

Cheng J, Randall AZ, Sweredoski MJ, Baldi P. 2005. SCRATCH: a protein structure and structural feature prediction server. Nucleic Acids Res 33:W72-76.

Coeytaux K, Poupon A. 2005. Prediction of unfolded segments in a protein sequence based on amino acid composition. Bioinformatics 21:1891-1900.

Dosztanyi Z, Csizmok V, Tompa P, Simon I. 2005. IUPred: web server for the prediction of intrinsically unstructured regions of proteins based on estimated energy content. Bioinformatics 21:3433-3434.

Galzitskaya OV, Garbuzynskiy SO, Lobanov MY. 2006. FoldUnfold: web server for the prediction of disordered regions in protein chain. Bioinformatics 22:2948-2949.

Ishida T, Kinoshita K. 2007. PrDOS: prediction of disordered protein regions from amino acid sequence. Nucleic Acids Res 35:W460-464.

Linding R, Jensen LJ, Diella F, Bork P, Gibson TJ, Russell RB. 2003. Protein disorder prediction: implications for structural proteomics. Structure 11:1453-1459.

Linding R, Russell RB, Neduva V, Gibson TJ. 2003. GlobPlot: Exploring protein sequences for globularity and disorder. Nucleic Acids Res 31:3701-3708.

Liu J, Rost B. 2003. NORSp: Predictions of long regions without regular secondary structure. Nucleic Acids Res 31:3833-3835.

Peng K, Vucetic S, Radivojac P, Brown CJ, Dunker AK, Obradovic Z. 2005. Optimizing long intrinsic disorder predictors with protein evolutionary information. J Bioinform Comput Biol 3:35-60.

Prilusky J, Felder CE, Zeev-Ben-Mordehai T, Rydberg EH, Man O, Beckmann JS, Silman I, Sussman JL. 2005. FoldIndex: a simple tool to predict whether a given protein sequence is intrinsically unfolded. Bioinformatics 21:3435-3438.

Shimizu K, Hirose S, Noguchi T. 2007. POODLE-S: web application for predicting protein disorder by using physicochemical features and reduced amino acid set of a position-specific scoring matrix. Bioinformatics 23:2337-2338.

Su CT, Chen CY, Hsu CM. 2007. iPDA: integrated protein disorder analyzer. Nucleic Acids Res 35:W465-472.

Vullo A, Bortolami O, Pollastri G, Tosatto SC. 2006. Spritz: a server for the prediction of intrinsically disordered regions in protein sequences using kernel machines. Nucleic Acids Res 34:W164-168.

Yang ZR, Thomson R, McNeil P, Esnouf RM. 2005. RONN: the bio-basis function neural network technique applied to the detection of natively disordered regions in proteins. Bioinformatics 21:3369-3376.
